# Streptococcus pneumoniae Meningitis Associated With Over-the-Counter Sinus Irrigation

**DOI:** 10.7759/cureus.8258

**Published:** 2020-05-24

**Authors:** James H Winegarner, Jeffrey Wittkopp

**Affiliations:** 1 Emergency Medicine, Brooke Army Medical Center, San Antonio, USA

**Keywords:** meningitis, pneumococcal, neti pot, naegleria fowleri, streptococcus pneumoniae, sinus rinse

## Abstract

We present the first reported case of *Streptococcus pneumoniae* bacterial meningitis that may be associated with use of an over-the-counter sinus irrigation. Sinus rinse or “Neti Pot” use is a common remedy for sinus congestion and is felt to be relatively safe. Given its widespread use, it is important to report possible associations with disease, in this case *Streptococcus pneumoniae* meningitis. A 50-year-old male with a history of sinusitis presented to the emergency department with a headache and altered mental status and was ultimately diagnosed with *Streptococcus pneumoniae* meningitis and sepsis. He had been using over-the-counter sinus rinses several times daily with distilled water. The patient had no radiographic evidence of contiguous spread or traumatic injury that would make him susceptible to direct cerebrospinal fluid infection. Inquiring about sinus irrigation use should be considered in patients with suspected meningitis. Emergency clinicians must consider meningitis in patients with Neti Pot use.

## Introduction

*Streptococcus pneumoniae* is a common pathogen causing meningitis [1,2]. Meningitis most commonly results from hematogenous spread, but can result from regional infection, head trauma, or recent surgery [3]. We describe a case of *S. pneumoniae* meningitis linked with a history of daily vigorous over-the-counter sinus rinse. He did not have typical radiographic findings suggestive of a regional infection or localized invasion into the central nervous system (CNS). His sinus infection and history of sinus rinse was suggestive of association between “Neti Pot” use and meningitis. Given the common use of sinus irrigation, it is valuable to identify possible risks of this intervention.

## Case presentation

**Figure 1:**
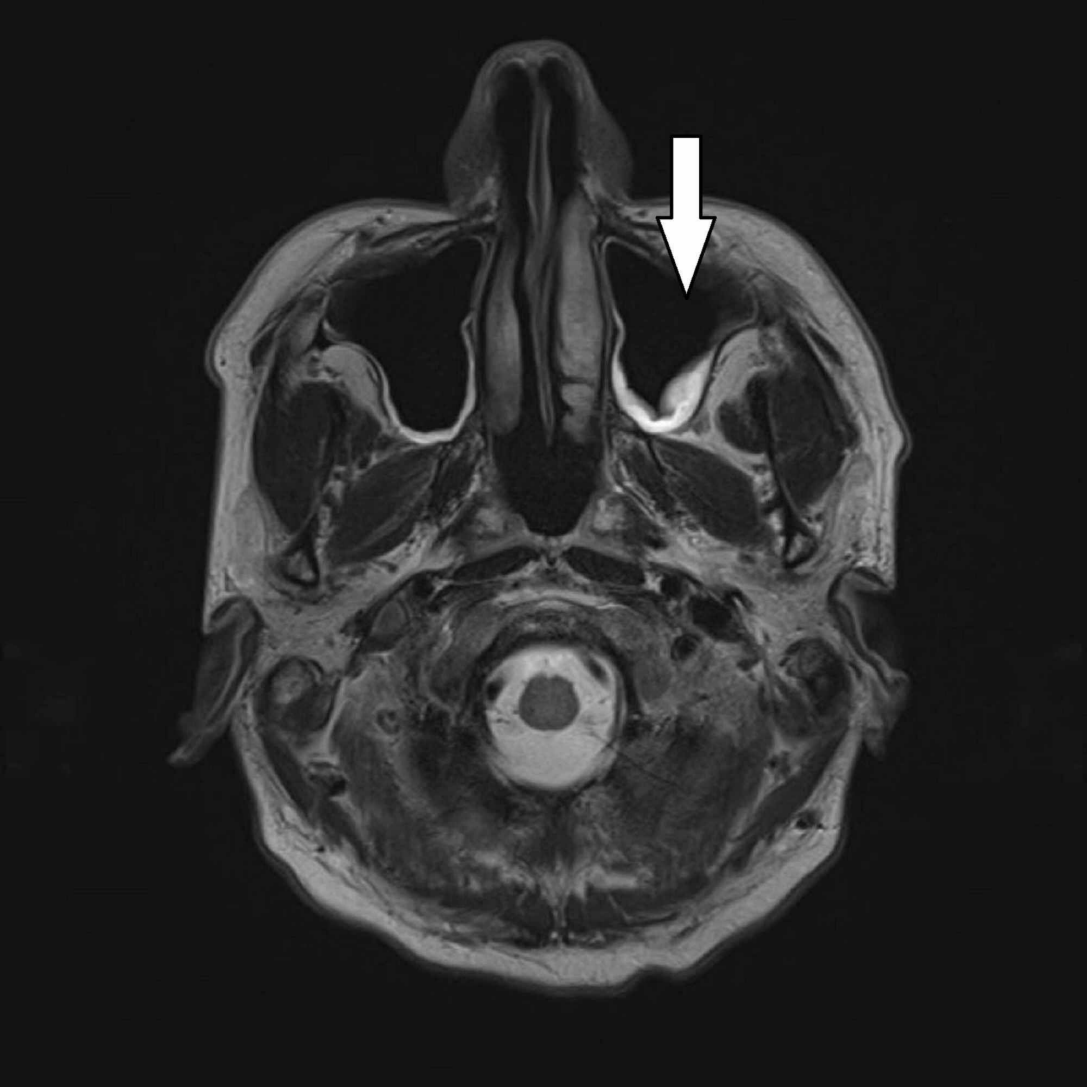
MRI demonstrating sinusitis

A 50-year-old male with a history of diabetes mellitus, hypertension, hyperlipidemia, and obstructive sleep apnea presented with a chief complaint of headache and altered mental status. His wife reported recent history of sinusitis and heavy use of over-the-counter sinus irrigation with distilled water as recommended on the package instructions. His physical examination on arrival was notable for fever, tachycardia, hypotension, and severe nuchal rigidity. CT of the head demonstrated pansinusitis, but no significant or destructive disease. Lumbar puncture (LP) revealed cerebrospinal fluid (CSF) white blood cell (WBC) count of 2424 per mm^3^. He was admitted to the intensive care unit (ICU) and treated empirically with ceftriaxone, vancomycin, ampicillin, and fluconazole. Fluconazole was added when initial CSF meningitis polymerase chain reaction (PCR) panel was positive for both *S. pneumoniae* and *Cryptococcus*. His fluconazole was switched to amphotericin B and flucytosine until subsequent India ink, cryptococcal antigen testing, and HIV were negative indicating the initial PCR was a false positive for *Cryptococcus*. CSF culture grew *S. pneumoniae*, and he was transitioned to ceftriaxone. MRI was obtained early in his course and demonstrated sinusitis and was otherwise normal (Figure 1). He had gradual improvement in his mental status and headache. Eventually he was discharged home, completed his antibiotic course, and had full resolution of his illness without sequelae.

## Discussion

*S. pneumoniae* is the most common cause of adult bacterial meningitis with the primary mechanism, including bacteremia seeding the CNS [1]. Bacterial meningitis is among the top infectious causes of death worldwide and when not fatal, may result in permanent neurological sequelae [2]. A presentation of headache, fever, neck stiffness, and altered mental status should prompt emergency physicians to obtain CSF and initiate empiric treatment for meningitis [3,4]. Laboratory evaluation of CSF should include gram stain, cell count, culture, glucose, protein, and PCR testing for causative organisms. CSF leukocyte count is the strongest finding, supporting a diagnosis of bacterial meningitis in recent studies [4]. CT scan of the brain can exclude other potential intracranial causes of headache and altered mental status and evaluate for evidence of increased intracranial pressure. CT before LP to evaluate the risk of herniation should be considered in patients with immunodeficiency, severe altered mental status, seizures, or focal neurological findings [3,4]. However, the decision to image with CT before LP should not delay initiation of treatment. MRI, if available, can be used to further evaluate for complications of meningitis, such as abscess or empyema, but is not a necessary part of the diagnosis [5]. 

Bacteria cause meningitis by invading the CNS and proliferating in the subarachnoid space. The CNS is well protected by the blood-brain barrier; however, bacteria must gain access through direct penetration (trauma, surgery, or severe localized infection such as mastoiditis) or hematogenous spread [2]. There have been rare cases of *Naegleria fowleri* primary amebic meningoencephalitis associated with sinus rinse, but literature review did not support any associations with sinus rinse and *S. pnuemoniae* meningitis [6,7]. The amebic route of entry to the CNS may be due to direct invasion through the olfactory nerve [7]. Bacterial entry into the CNS does not appear to follow the same route as *N. fowleri*. Bacterial hematogenous seeding appears to begin with mucosal colonization, followed by lymphatic and intravascular invasion, exposure to brain vessel endothelium, and transmigration across this layer [2]. Once bacteria have crossed the brain endothelium, microbes may spread in the perivascular space around penetrating blood vessels throughout the brain [5].

Our patient did not have MRI or CT evidence of penetrating trauma, nor did he have significant upper respiratory infection or pneumonia as a source of systemic infection. There are several possible explanations for his source of meningitis. His chronic pansinusitis may have been a source of mucosal colonization with *S. pnuemoniae*. Daily vigorous nasal irrigation could have introduced the bacteria into the bloodstream, or the Neti Pot bottle may have been colonized with *S. pnuemoniae*. While causality is impossible to prove, the sinus rinse may be associated with meningitis and should be considered as a risk factor for meningitis.

## Conclusions

Sinus irrigation may be associated with bacterial meningitis, and further studies are required to determine if sinus rinse use is truly a risk factor for bacterial meningitis. Neti Pot use is commonly recommended for patients with viral upper respiratory infections, and it is valuable to identify if this recommendation increases patient risk for bacterial meningitis. Physicians treating meningitis should consider sinus rinse use as a potential risk factor for meningitis. 
